# Smartphone-Based Chemiluminescence Glucose Biosensor Employing a Peroxidase-Mimicking, Guanosine-Based Self-Assembled Hydrogel

**DOI:** 10.3390/bios13060650

**Published:** 2023-06-14

**Authors:** Donato Calabria, Andrea Pace, Elisa Lazzarini, Ilaria Trozzi, Martina Zangheri, Massimo Guardigli, Silvia Pieraccini, Stefano Masiero, Mara Mirasoli

**Affiliations:** 1 Department of Chemistry “Giacomo Ciamician”, Alma Mater Studiorum—University of Bologna, Via Francesco Selmi 2, I-40126 Bologna, Italy; donato.calabria2@unibo.it (D.C.); andrea.pace7@unibo.it (A.P.); elisa.lazzarini6@unibo.it (E.L.); ilaria.trozzi2@unibo.it (I.T.); martina.zangheri2@unibo.it (M.Z.); massimo.guardigli@unibo.it (M.G.); silvia.pieraccini@unibo.it (S.P.); stefano.masiero@unibo.it (S.M.); 2 Interdepartmental Centre for Industrial Aerospace Research (CIRI AEROSPACE), Alma Mater Studiorum—University of Bologna, Via Baldassarre Canaccini 12, I-47121 Forlì, Italy; 3 Interdepartmental Centre for Industrial Agrofood Research (CIRI AGRO), Alma Mater Studiorum—University of Bologna, Via Quinto Bucci 336, I-47521 Cesena, Italy; 4 Interdepartmental Centre for Industrial Research in Advanced Mechanical Engineering Applications and Materials Technology (CIRI MAM), Alma Mater Studiorum—University of Bologna, Viale Risorgimento 2, I-40136 Bologna, Italy; 5 Interdepartmental Centre for Industrial Research in Renewable Resources, Environment, Sea and Energy (CIRI FRAME), Alma Mater Studiorum—University of Bologna, Via Sant’Alberto 163, I-48123 Ravenna, Italy

**Keywords:** hydrogel, guanosine, enzyme mimic, G-quadruplex, supramolecular chemistry, chemiluminescence, biosensor, hydrogen peroxide, glucose, point of care

## Abstract

Chemiluminescence is widely used for hydrogen peroxide detection, mainly exploiting the highly sensitive peroxidase-luminol-H_2_O_2_ system. Hydrogen peroxide plays an important role in several physiological and pathological processes and is produced by oxidases, thus providing a straightforward way to quantify these enzymes and their substrates. Recently, biomolecular self-assembled materials obtained by guanosine and its derivatives and displaying peroxidase enzyme-like catalytic activity have received great interest for hydrogen peroxide biosensing. These soft materials are highly biocompatible and can incorporate foreign substances while preserving a benign environment for biosensing events. In this work, a self-assembled guanosine-derived hydrogel containing a chemiluminescent reagent (luminol) and a catalytic cofactor (hemin) was used as a H_2_O_2_-responsive material displaying peroxidase-like activity. Once loaded with glucose oxidase, the hydrogel provided increased enzyme stability and catalytic activity even in alkaline and oxidizing conditions. By exploiting 3D printing technology, a smartphone-based portable chemiluminescence biosensor for glucose was developed. The biosensor allowed the accurate measurement of glucose in serum, including both hypo- and hyperglycemic samples, with a limit of detection of 120 µmol L^−1^. This approach could be applied for other oxidases, thus enabling the development of bioassays to quantify biomarkers of clinical interest at the point of care.

## 1. Introduction

The monitoring of health-related biomarkers at the point of care (POC) by means of simple, cost-effective, and easy-to-use diagnostic tests could revolutionize screening procedures, reducing the incidence of chronic pathologies, and improve patient survival rates and people’s life quality. In particular, the availability of instrument-free and rapid diagnostic devices is considered a fundamental step to reduce healthcare costs and to enlarge the screening scale, thus providing a real breakthrough in the diagnostic field [1]. A recent major development for the decentralization and democratization of clinical laboratory tests has been the combination of smartphones and (bio)sensors [2]. To this end, optical biosensors are particularly advantageous because their interfacing with smartphones can be quite simple and straightforward, while electrochemical ones require dedicated equipment and a power supply for signal generation and measurement at the electrodes [2,3].

Hydrogen peroxide is involved in many physiological processes, such as metabolic processes, apoptosis, and immune-cell activation [4,5,6,7]. Owing to its roles as an oxidative stress marker, defense agent, and aging promoter [8], it is recognized as a crucial biomarker of various diseases including diabetes [9], cancer [10], Parkinson’s, cardiovascular, Alzheimer’s, and neurodegenerative disorders [11]. Moreover, H_2_O_2_ is produced by oxidase enzymes (e.g., glucose oxidase, alcohol oxidase, cholesterol oxidase, lactate oxidase, and glutamate oxidase). This allows us to develop bioassays relying on H_2_O_2_ detection for the quantification of such enzymes, as well as of their substrates [12]. Therefore, the development of (bio)sensors for H_2_O_2_ based on peroxidase activity is an active research field, finding applications in medical diagnostics, clinical research, food chemistry, environmental investigations, and industrial process monitoring [13,14,15,16,17,18,19,20,21].

The use of natural enzymes in biosensor technology has some drawbacks, such as their limited stability and high cost, especially when multiple enzymes are required [1]. However, advances in materials science and nanotechnology have led to several strategies for obtaining synthetic enzymes as substitutes for natural ones [22,23]. Even though synthetic enzymes sometimes show reduced catalytic activity with respect to their natural counterparts, they offer high stability in the surrounding environment, low cost, simple synthesis, easy chemical modification, long-term storage without a decrease in catalytic activity, and the possibility to recover the enzyme after reaction. All these properties make them promising candidates as non-biological recognition elements in electrochemical and optical biosensors [24,25,26,27].

**Figure 1:**
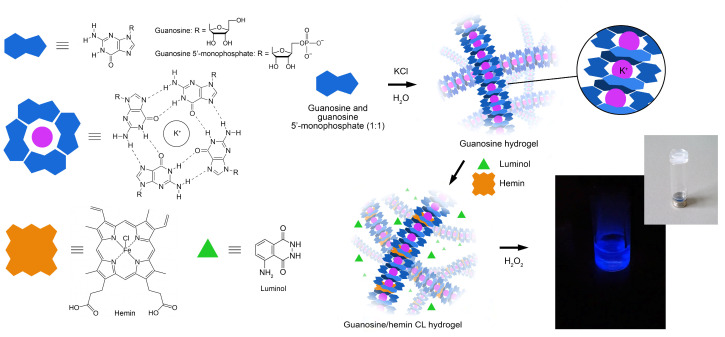
Scheme of the formation of the guanosine/hemin CL hydrogel, by supramolecular self-assembly of guanosine and guanosine 5′-monophosphate in the presence of K^+^, hemin, and luminol.

In recent years, hydrogels have gained great interest in the development of biosensors. Taking advantage of their ability to incorporate foreign substances while preserving a benign environment for biosensing events, hydrogels have been exploited as functional materials in biosensing [28,29,30,31]. Their 3D porous structure implies a wide surface area of the material, allowing the loading of large amounts of recognition elements (ranging from small molecules to proteins and even cells), which remain easily accessible to substrates or analytes. Furthermore, hydrogels provide a biocompatible environment thanks to their flexible and highly water-swellable nature. Indeed, the preservation of the native structure of biomolecules is a crucial requirement for feasibility, specificity, and sensitivity in biosensing applications. DNA represents an ideal candidate for supramolecular gelation because of its reversible hybridization reaction via non-covalent interactions [32,33]. However, the large amount of material required for their preparation makes DNA-based hydrogels expensive, and the utilization of nucleosides and their analogs as alternative starting materials becomes an effective solution [34]. Recently, the self-assembly reactions of guanosine (G) and its derivatives served as an inspirational approach for the design of functional soft materials displaying enzyme-like catalytic activity [35]. In this approach, the G-quartets (G4) formed by the self-assembly of four guanosine bases via Hoogsteen-type hydrogen bond networks produced nanofibrous G-quadruplex structures in the presence of metal ions, such as K^+^ (Figure 1). The ability of guanosine derivatives to form stable supramolecular architectures has been widely studied by some of us [36,37]. In particular, the assembly of the G4 subunits offers unique possibilities for generating functional materials such as gels, cross-linked polymers, and synthetic ion channels [35,36,37,38,39,40,41]. In addition, the incorporation of hemin into G4 columnar structures gives rise to a synthetic enzyme showing peroxidase-like activity and good biocompatibility [35,41]. Consequently, G4-based enzyme-like hydrogels are excellent functional materials for biosensor development.

Herein, we propose a smartphone-based chemiluminescence (CL) biosensor for the detection of H_2_O_2_ and glucose. Glucose is an important source of carbon and energy and a biomarker of many diseases. Glucose monitoring in blood is of great significance in clinical practice, particularly in the diagnosis and management of diabetes. To this end, the development of improved biosensors for measuring blood glucose levels at the POC is an active research field, aiming at providing a significant improvement in the management of diabetes [42,43,44,45].

In this paper, a binary guanosine hydrogel prepared using a mixture of guanosine and guanosine 5′-monophosphate in the presence of K^+^ ions [46,47] is loaded with a CL reagent (luminol) and a catalytic cofactor (hemin), to produce a functional material showing peroxidase-like activity to the CL reaction of luminol with H_2_O_2_. The hydrogel is then functionalized with glucose oxidase (GOx) enzyme to enable glucose biosensing: the hydrogen peroxide produced by GOx (or, in principle, by any other oxidase enzyme) reacts, in the presence of the self-assembled guanosine/hemin gel mixture, with luminol to produce photon emission (Figure 1). The biosensor takes advantage of both the features of CL detection, offering high detectability and amenability to miniaturization [15,17,21,48], and of the 3D porous structure of hydrogel, as providing increased stability to incorporated enzymes [42,49]. To provide assay POC applicability, photon emission was detected by means of a portable device employing a smartphone’s CMOS (complementary metal oxide semiconductor) camera for the detection of the CL emission.

## 2. Materials and Methods

### 2.1. Reagents

Hemin, luminol sodium salt, glucose oxidase (GOx) enzyme from *Aspergillus niger*, human serum albumin (HSA), glucose, glucose-6-phosphate, sucrose, galactose, trehalose, mannose, hydrogen peroxide, and guanosine 5′-monophosphate disodium salt hydrate were purchased from Merck KGaA (Darmstadt, Germany). Guanosine was purchased from TCI (Portland, OR).

The following buffers, prepared in Milli-Q Plus ultra-pure water, were used in the preparation of the hydrogel and in the enzymatic assays: carbonate buffer (0.1 mol L^−1^ carbonate buffer, pH 10.8), phosphate buffered saline (PBS, 0.01 mol L^−1^ phosphate buffer containing 137 mmol L^−1^ NaCl and 2.7 mmol L^−1^ KCl, pH 7.4), Tris buffer (50 mmol L^−1^ Tris buffer containing 100 mmol L^−1^ NaCl and 2.7 mmol L^−1^ KCl, pH 7.4).

The single-stranded DNA (ssDNA) designed DNAzyme was obtained by mixing a ssDNA sequence (5′-TTTTGGGTGGGTTGGGTGGGT-3′) purchased from Integrated DNA Technologies (Coralville, IA, USA) with hemin in Tris buffer to reach a final 200 mmol L^−1^ concentration for both species and incubating the mixture for 30 min at room temperature.

The CL measurements in microplates were performed in black 96-well microtiter plates using a Varioskan LUX microtiter plate luminometer (Thermo Scientific, Waltham, MA, USA). The enzymatic glucose colorimetric assay (Glucose Colorimetric Detection Kit) in the 96-well microplate format used as a reference method for assessing glucose concentration of real samples was bought from Life Technologies Corporation (Thermo Fisher Scientific, Frederick, MD, USA).

### 2.2. Preparation of the Guanosine/Hemin CL Hydrogel

A binary 100 mmol L^−1^ guanosine hydrogel was prepared by following a previously published procedure with modifications [46]. Guanosine (0.25 mmol) and guanosine 5′-monophosphate disodium salt (0.25 mmol) were added with 5 mL of 50 mM aqueous KCl in a glass tube. The vial was heated at 95 °C in a water bath for 10 min. The resulting clear solution was then left to cool down at room temperature in the dark and a transparent gel formed in 15–20 min. After 1h, the gel phase was heated again at 95 °C for 5 min, then 200 μL of hemin (100 μmol L^−1^ in carbonate buffer) and 500 μL of luminol (10 mmol L^−1^ in 0.1 M NaOH) were sequentially added to the hot isotropic solution [50]. The thus-obtained guanosine/hemin CL hydrogel (Figure 1) was stored for one night at room temperature in the dark before further use. A pH of 9.2 was detected in the final gel phase.

### 2.3. Measurement of H_2_O_2_ and Glucose in the Microtiter Plate Format

For the measurement of H_2_O_2_ in the microtiter plate format, the guanosine/hemin CL hydrogel was heated to 80 °C on a heating plate to be converted to the liquid state, then dispensed into the wells of a microplate (80 μL for each well) and allowed to cool to room temperature. To generate a H_2_O_2_ calibration curve, 100 μL of H_2_O_2_ solutions in PBS buffer at different concentrations (ranging from 0.1 µmol L^−1^ to 1.0 mmol L^−1^) or PBS buffer for the blank were added to each well and the CL emission was immediately measured by the microtiter plate luminometer. The emission was recorded for 1h to obtain the CL kinetic profiles for each well, then the analytical CL signals were calculated as the area under the curves. For measuring glucose, the GOx enzyme-loaded CL hydrogel was prepared as follows. The guanosine/hemin CL hydrogel was heated to about 90 °C, then, upon letting it cool down to about 60 °C, a 5 mg mL^−1^ (500 U mL^−1^) GOx enzyme solution in PBS was added in a hydrogel:enzyme solution in a 8:1 (*v/v*) ratio. Following this, the mixture was immediately dispensed into the wells of a black 96-well microtiter plate (90 μL for each well) and allowed to cool to room temperature. The calibration curve was generated using glucose standard solutions in PBS buffer (0.1 µmol L^−1^–1.0 mmol L^−1^ concentration range) or PBS buffer as blank.

### 2.4. Smartphone-Based CL Detection with 3D Printed Device

To enable POC applicability of the hydrogel-based biosensor, a portable analytical device was developed allowing the measurement of the CL signal using a Samsung Galaxy S20 Plus smartphone (Samsung Group, Seoul, Republic of Korea). The analytical device consisted of two 3D printed components: a disposable analytical cartridge and a dark box. Both components were designed using Fusion 360 CAD software (Autodesk Inc., San Rafael, CA, USA) and produced in black resin employing a commercial Form 2 stereolithography (SLA) 3D printer (Formlabs Inc., Somerville, MA, USA). The analytical cartridge contained four wells (diameter 8 mm, volume 250 µL), thus providing a convenient portable assay format for a limited number of standard solutions and/or samples. To perform the CL measurement, the cartridge was inserted into the dark box that, after being connected to the smartphone, eliminated the interference of the ambient light in the measurement, also assuring the reproducible positioning of the analytical cartridge in front of the smartphone’s CMOS camera and the correct focal distance for acquisition of CL images.

To produce the analytical cartridges, the GOx enzyme-loaded CL hydrogel, prepared as described in Section 2.3, was dispensed into the wells of the cartridge (90 μL for each well) and allowed to cool to room temperature. Analytical cartridges could be prepared in advance, sealed in plastic bags, and stored in the dark at +4 °C for up to four weeks.

To perform the measurement, the bag containing the analytical cartridge was retrieved from cold stowage and allowed to reach room temperature. Following this, the cartridge was removed from the bag and 100 µL of three glucose standard solutions in PBS (0.5 mmol L^−1^, 1.5 mmol L^−1^, and 2.5 mmol L^−1^) and the sample were dispensed in the wells. The cartridge was inserted into the dark box, and after a 30-min incubation the CL emission was measured. The CL image of the cartridge was acquired with the following parameters: sensitivity ISO (International Organization for Standardization) 3200 and integration time 60 s. The Android Camera FV-5 app, available on Google Play, was employed (other camera apps enabling long exposure times and automated acquisition of image sequences could be also employed).

### 2.5. Real Sample Analysis

The applicability of the smartphone-based biosensor for the analysis of real samples was assessed by analyzing glucose-spiked artificial serum samples. Artificial serum (containing NaCl 6.8 mg mL^−1^, CaCl_2_ 0.2 mg mL^−1^, KCl 0.4 mg mL^−1^, MgSO_4_ 0.1 mg mL^−1^, NaHCO_3_ 2.2 mg mL^−1^, Na_2_HPO_4_ 0.126 mg mL^−1^, NaH_2_PO_4_ 0.026 mg mL^−1^, and HSA 35 g L^−1^) was prepared following a published procedure with slight modifications [51,52] and spiked with known amounts of glucose (in the range 0.5–10.0 mmol L^−1^). The samples were diluted 1:4 (*v/v*) with PBS buffer prior to the analysis with both the biosensing device and the reference enzymatic glucose colorimetric assay.

### 2.6. Data Analysis

The freeware ImageJ software (v.1.53 h, National Institutes of Health, Bethesda, MD, USA) was employed for the quantitative analysis of the CL images. First, regions of interest (ROIs) corresponding to the well areas of the disposable cartridge of the biosensor were defined, then for each image the CL signals were evaluated by integrating the CL emissions over the ROI areas. Data graphing and analysis were performed using GraphPad Prism (version 8.0, GraphPad Software, Inc., La Jolla, CA, USA).

## 3. Results and Discussion

### 3.1. Design of the G-Quadruplex Hydrogel-Based Biosensor

In this work, a self-assembled nanofibrous G4-based hydrogel was exploited for developing a smartphone-based CL biosensor for detecting H_2_O_2_ and glucose (through its GOx-catalyzed oxidation reaction) at the POC. The supramolecular gel phase, exhibiting thermal reversible sol-gel transition, consisted of K^+^ stabilized G-quadruplex structures [46,53] and showed, upon hemin incorporation, peroxidase-like activity to the oxidation of luminol by H_2_O_2_. Owing to its nanofibrous entangled structure, the hydrogel constituted an optimal matrix for loading enzymes, providing increased enzyme stability and catalytic activity even in highly alkaline and oxidizing conditions [49,52]. Indeed, the alkaline pH of the hydrogel used in the biosensor (pH ~ 9) was optimal for obtaining intense CL emission by the luminol-H_2_O_2_ system, but it was far from the pH value providing the maximum activity of GOx from *Aspergillus niger* (pH 5.5) [54]. The ability of hydrogel to preserve enzyme activity even in an alkaline environment was very advantageous, as it enabled us to simplify the analytical protocol by carrying out all the reactions in a single compartment and at a basic pH. As previously reported, the compatibility of the different reaction environments is a common drawback encountered in the use of coupled enzyme reactions, for which, in most cases, the two enzyme-catalyzed reactions must be performed in sequence, each in its optimal milieu [17,21]. The proposed hydrogel displayed additional positive features, such as simple, low-cost, and rapid synthesis [46], thermo-reversible gelation, environmental friendliness [50], good biocompatibility [55], and inertness towards the analyte and the reagents. In addition, it was highly transparent in the visible range [50] and permeable to hydrogen peroxide and glucose by diffusion, thus providing a uniform and reproducible light emission in the whole hydrogel volume.

### 3.2. G-Quadruplex Hydrogel Performance for H_2_O_2_ Quantitative Detection

**Figure 2:**
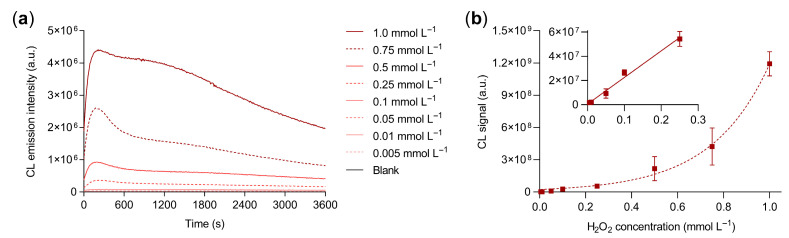
(**a**) CL kinetic profiles obtained for different concentrations of H_2_O_2_; (**b**) CL calibration curve for the quantification of H_2_O_2_ obtained from the quantitative analysis of the CL kinetic profiles. The inset of (**b**) shows the linear part of the calibration curve (i.e., between 5 and 250 µmol L^−1^ of H_2_O_2_). The equation of the linear calibration curve is Y = (2.2 × 10^8^ ± 4 × 10^7^)X + (6 × 10^5^ ± 5.4 × 10^6^), R^2^ = 0.98, where Y is the CL signal and X is the concentration of H_2_O_2_ in mmol L^−1^. Each datum is the mean ± SD of three independent experiments; a.u.: arbitrary units.

To ensure the optimal analytical conditions, the CL response of the hydrogel in the presence of different concentrations of H_2_O_2_ (from 0.5 µmol L^−1^ to 1.0 mmol L^−1^) was evaluated in the 96-well microtiter plate format (Figure 2a). The calibration curve (Figure 2b) showed a CL signal increase with the amount of H_2_O_2_. A good linear correlation between the CL signal and the concentration of H_2_O_2_ (R^2^ = 0.98) was found in the 5–250 µmol L^−1^ concentration range and the limit of detection (calculated as the H_2_O_2_ concentration corresponding to the blank signal plus three times its standard deviation) was 7.0 µmol L^−1^ (corresponding to 700 pmol of H_2_O_2_). The working range of the calibration curve appears to be adequate for distinguishing between physiological and pathological H_2_O_2_ levels in plasma. Indeed, while reference values are still under debate in the scientific community, a recent literature survey [56] suggested physiological ranges below 10 µM and higher levels (30–50 µM) in certain pathological conditions, such as inflammatory diseases. Overall, these results confirmed the enzyme-like activity of the guanosine/hemin CL hydrogel towards the oxidation of luminol by H_2_O_2_.

**Figure 3:**
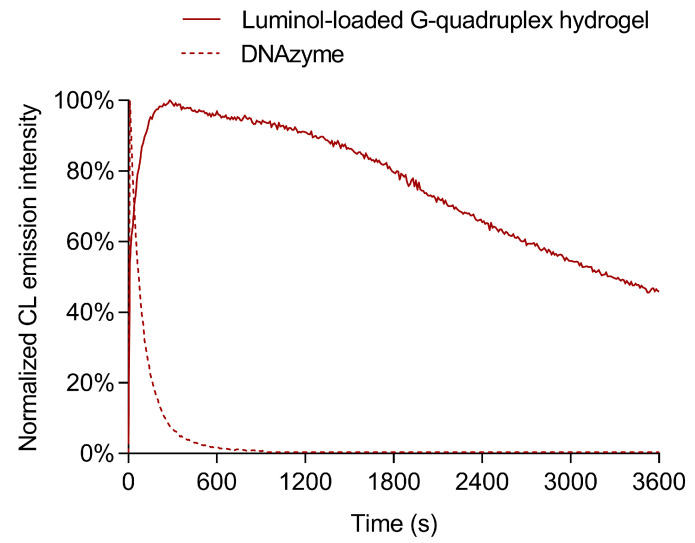
Comparison between the kinetic profiles of the CL emissions obtained with the luminol-loaded G-quadruplex hydrogel (upon addition of hydrogen peroxide) and a reference ssDNA designed DNAzyme (upon addition of luminol and hydrogen peroxide). To facilitate the comparison of the kinetic profiles, each profile has been normalized to its maximum intensity.

To further investigate the CL response of the hydrogel to H_2_O_2_, we compared its CL kinetic profile to that of a ssDNA designed hemin/G-quadruplex DNAzyme. Indeed, hemin/DNA-based G-quadruplex structures prepared employing guanine-rich ssDNA sequences are widely used as peroxidase-like DNAzymes in CL biosensors [57]. As shown in Figure 3, upon addition of a CL cocktail containing 10 mmol L^−1^ luminol and 1.0 mmol L^−1^ H_2_O_2_ in carbonate buffer, the ssDNA designed DNAzyme displayed a fast photon emission kinetics, in which the CL emission reached its maximum intensity in few seconds, then decayed to background signal in about ten minutes. Therefore, the measurement of its CL emission would require automatic sample injection, which complicates the design of a portable analytical device. Conversely, the guanosine/hemin CL hydrogel provided glow-type, long-lasting photon-emission kinetics and the maximum CL was observed several minutes after the addition of the H_2_O_2_ solution. The ability to stabilize the CL signal over time by means of slow diffusion-controlled penetration of H_2_O_2_ into the gel facilitated the design of simple and cheap smartphone-based biosensing devices. In addition, the overall photon emission observed with the guanosine/hemin CL hydrogel was at least one order of magnitude higher with respect to that of the ssDNA designed DNAzyme, when tested with the same amounts of luminol and H_2_O_2_, thus suggesting a higher efficiency for the luminol/H_2_O_2_ CL reaction. It must be also noted that an increased structural stability was expected for the guanosine-based hydrogel due to the absence of lateral loops composed of one or more nucleotides that have been reported to modify its topology upon conformational changes [58]. Deeper investigation of these aspects will be the subject of future studies.

### 3.3. G-Quadruplex Hydrogel Performance for Glucose Quantitative Detection

**Figure 4:**
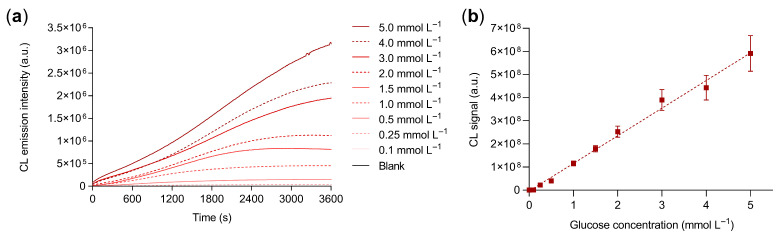
(**a**) CL kinetic profiles obtained for different concentrations of glucose and (**b**) calibration curve for the quantification of glucose obtained from the quantitative analysis of the CL kinetic profiles. The equation of the linear calibration curve is Y = (1.19 × 10^8^ ± 8 × 10^6^)X + (−4 × 10^6^ ± 2.0 × 10^7^), R^2^ = 0.99, where Y is the blank-subtracted CL signal and X is the glucose concentration in mmol L^−1^. Each datum is the mean ± SD of three independent experiments; a.u.: arbitrary units.

The 3D porous structure of the guanosine/hemin CL hydrogel was exploited to incorporate GOx enzyme, thus enabling the quantitative detection of glucose. Upon sample addition, glucose diffuses into the hydrogel and is oxidized by the enzyme with production of H_2_O_2_, which then triggers photon emission. Figure 4a shows the CL kinetic profiles recorded upon addition of standard glucose solutions in PBS in the range 0.1 to 5.0 mmol L^−1^. The kinetics are slower than those obtained in the presence of H_2_O_2_, which is consistent with the two-step nature of the CL production process (i.e., the intensity of CL emission now also depends on the rate of glucose oxidation to H_2_O_2_). A linear calibration curve was obtained over the whole calibration concentration range (Figure 4b) with an estimated limit of detection of 50 µmol L^−1^ (corresponding to 5 nmol of glucose).

### 3.4. Smartphone-Based Glucose Biosensing Device

The hydrogel-based glucose bioassay was integrated into a portable device, taking advantage of the peculiar characteristics of CL measurements, such as high sensitivity (i.e., high signal-to-noise ratio) and simplicity of instrumental requirements (no light sources, wavelength selection systems, specific geometry of the sample container are required). Moreover, thanks to the intense and long-lasting photon emission, the CL measurement could be successfully performed by the built-in CMOS camera of a commercial smartphone.

**Figure 5:**
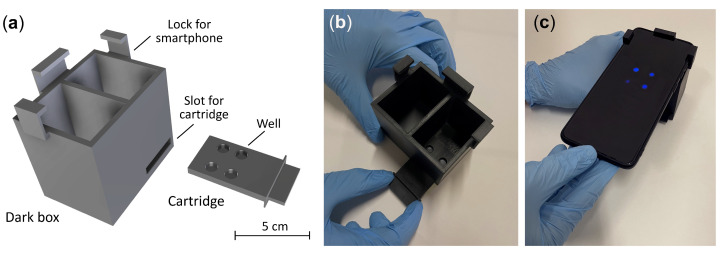
(**a**) Scheme of the 3D-printed cartridge and the dark box used for the glucose bioassay employing smartphone-based CL detection; (**b**,**c**) Images taken during the measurement of the CL emission in a cartridge containing three glucose standards and an unknown sample.

Stereolithography (SLA) 3D printing was exploited to produce device components with a complex shape, which includes an analytical cartridge with four wells to be loaded with the GOx enzyme-loaded CL hydrogel (90 µL per well) and a mini dark box (Figure 5). In the assembled device, the dark box has the role of positioning the cartridge at the correct distance from the smartphone CMOS camera and, upon being connected to the smartphone, to prevent interference from ambient light during the measurement. A critical issue in the design of smartphone-based optical biosensors is the distance between the smartphone’s built-in camera and the detection zone. Modern smartphones’ built-in camera technology enables focusing at as close as a 4–5 cm distance, or even less if the smartphone is equipped with a macro camera. Exploiting this feature, very compact devices can be designed without the need to add focusing lenses [17,59]. The app used for image acquisition should also be considered, since in addition to the app provided by the producer, many others (either free or commercial) are available. The selection should be mainly based on the maximum image exposure time allowed by the software. Indeed, to acquire the weak CL emission, the sensitivity of the camera should be improved either by lengthening image exposure time, or by increasing sensor sensitivity (i.e., using higher ISO numbers) [17,59]. Nevertheless, longer exposure time (e.g., up to 60 s) should be preferred to higher sensor sensitivity because the latter also increases the noise in the acquired CL image.

**Figure 6:**
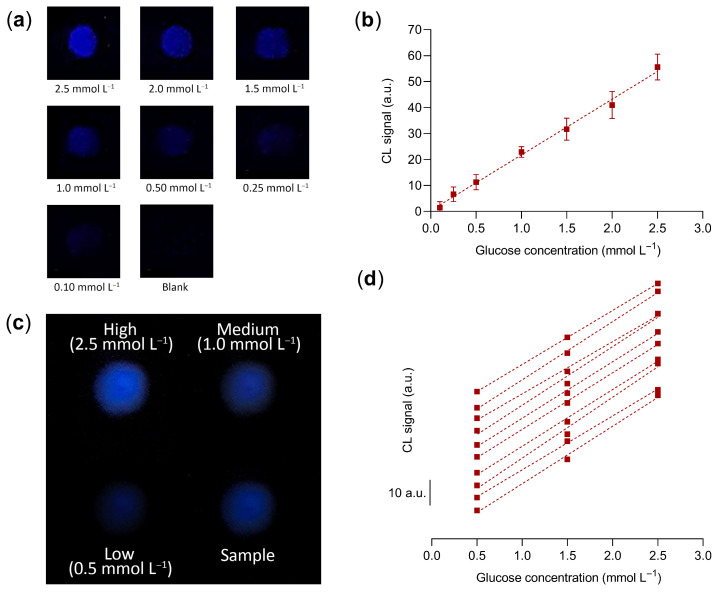
(**a**) CL images obtained for the analysis of solutions containing different concentrations of glucose using the smartphone-based assay device and (**b**) calibration curve for the quantification of glucose obtained from the quantitative analysis of the CL images. The equation of the linear calibration curve is Y = (21.4 ± 1.7)X + (0.3 ± 1.4), R^2^ = 0.99, where Y is the blank-subtracted CL signal and X is the glucose concentration in mmol L^−1^. Each datum is the mean ± SD of three independent experiments. (**c**) CL image of a cartridge with the three glucose standard solutions and a sample acquired during the assay. (**d**) Set of ten three-point calibration curves for the quantification of glucose obtained from the quantitative analysis of the CL images of different cartridges (the curves have been shifted along the Y axis to avoid overlapping). The slopes of the curves were in the range 20.2–23.5 (mean value 21.8 ± 1.0), while the Y-intercepts ranged from −2.3 to 1.5 (mean value −0.3 ± 1.2); a.u.: arbitrary units.

Figure 6a shows representative CL images of the wells of the analytical cartridge acquired upon addition of glucose standard solutions in PBS in the range 0.1–2.5 mmol L^−1^ of glucose. The corresponding calibration curve (Figure 6b) was linear in the whole concentration range and the estimated limit of detection was 120 µmol L^−1^ of glucose (i.e., about 2.5 times the value found in the 96-well microtiter analytical format).

Since the number of samples that can be analyzed in a single cartridge is limited, an analytical procedure that relies on a calibration curve obtained by analyzing several standards in replicate could be only performed using many analytical cartridges. To perform the assay in a single cartridge, we evaluated a simpler procedure that requires the analysis in the same cartridge of the sample and of three glucose standards, i.e., corresponding to low (0.5 mmol L^−1^), intermediate (1.5 mmol L^−1^), and high (2.5 mmol L^−1^) glucose concentrations (a blank measurement was not performed, thus avoiding the requirement of an additional blank well in the cartridge). A three-point linear calibration curve was obtained using the standards’ CL signals, then the sample’s concentration of glucose was evaluated by interpolation of its CL signal on the calibration curve. To prove the feasibility of the three-standard calibration approach, we used analysis of covariance (ANCOVA) [60,61] to compare a set of ten three-point calibration curves obtained in different cartridges (Figure 6d) with the calibration curve of Figure 6b (Figure 6c shows the CL image of a cartridge acquired during the assay). The comparison test showed that, for each three-point curve, both slope and intercept were not significantly different (*p* > 0.05) from those of the reference calibration curve. This demonstrated that reliable calibration curves can be generated in the analytical cartridge using a limited number of standards. In addition, as shown in Section 3.6, using the three-point calibration curve a good accuracy was obtained in the analysis of real samples.

### 3.5. Assay Selectivity

The selectivity of the hydrogel-based CL glucose biosensor was verified by comparing the response to glucose to those obtained for interferents such as glucose 6-phosphate, sucrose, galactose, trehalose, and mannose (glucose and interferents were assayed at a concentration of 1.25 mmol L^−1^).

**Figure 7:**
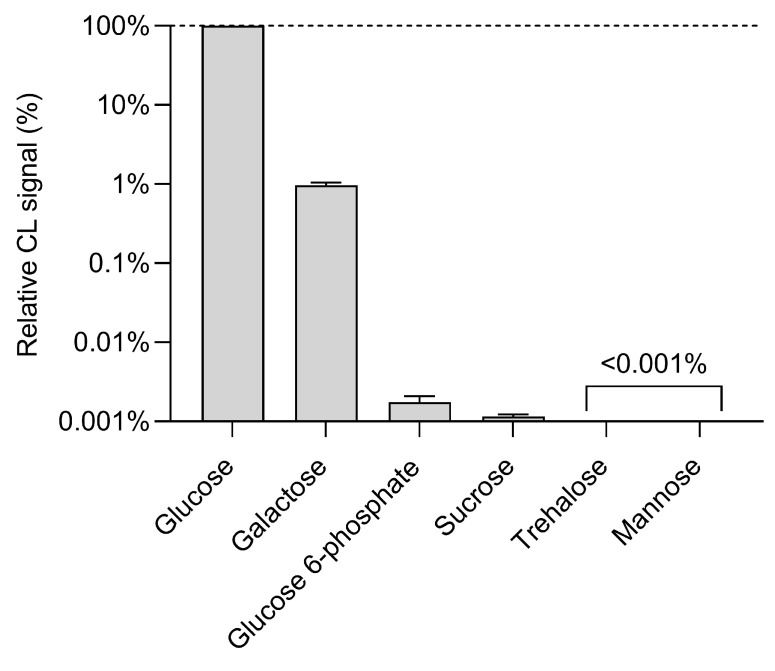
Comparison of the CL signals obtained by analyzing 1.25 mmol L^−1^ solutions in PBS of glucose and potentially interferent sugars (the CL signals obtained for the interferents have been normalized to that of glucose). Each datum is the mean ± SD of three independent experiments.

As shown in Figure 7, there is no significant interference by the other sugars considered. The highest cross-reactivity (with a CL signal corresponding to about 0.9% of that of glucose) was observed for galactose, while for the other interferents the CL signals were almost negligible, thus demonstrating the high specificity of the biosensor for glucose.

### 3.6. Application to Real Samples

To evaluate the assay accuracy of the smartphone-based biosensor on real samples, recovery experiments were performed by analyzing artificial serum samples spiked with known amounts of glucose. The samples, spiked with glucose concentrations ranging from 0.5 to 10.0 mmol L^−1^, were analyzed in 1:4 (*v/v*) dilution with PBS to comprise the assay calibration range (in addition, the samples’ dilution with PBS allowed us to reduce a possible matrix effect). The spiked concentrations were selected to comply with the physiological levels of glucose in blood, also including hypoglycemic and hyperglycemic samples (blood glucose levels in fasting healthy subjects are in the range 70–130 mg dL^−1^, corresponding to 3.9–7.1 mmol L^−1^ [62]). The assay results were compared to those obtained with a commercial enzymatic glucose colorimetric assay in the standard 96-well microtiter plate format (Table 1). The recovery values (in the range 92–110%) and the good correlation observed between the results of both methods demonstrates the ability of the assay to provide accurate quantification of glucose in serum samples, either at physiological or hypo- and hyperglycemic levels.

### 3.7. Stability of the Glucose Biosensor

The guanosine/hemin CL hydrogel was macroscopically stable for at least one year, as confirmed by a tube-inversion test and CL emission intensity, when stored at 4 °C in the dark and in a sealed container to avoid water evaporation. The stability of the cartridges containing the GOx enzyme-loaded CL hydrogel was evaluated by measuring the CL signal obtained upon addition of glucose standard solutions using cartridges stored for various times in sealed plastic bags at +4 °C and in the dark. The response of the biosensor was maintained for at least four weeks (i.e., after a four-week storage, the CL signal decreased not more than 10%), showing, as previously reported [52], the ability of the guanosine-based hydrogel to preserve enzyme activity.

### 3.8. Hydrogel 3D Printing

**Figure 8:**
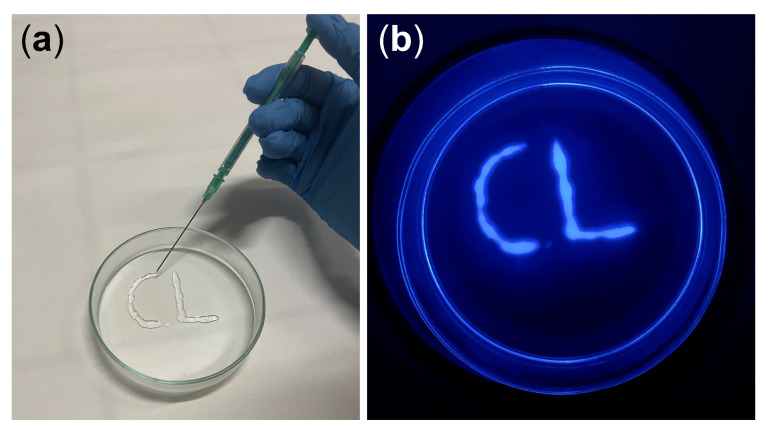
(**a**) 3D printing of the guanosine/hemin CL hydrogel with a syringe by exploiting its thixotropic property. (**b**) CL image of the 3D-printed hydrogel upon addition of H_2_O_2_.

Guanosine-derived hydrogels have been shown to possess good thixotropic properties [41,55] and consequently they can be used as injectable materials [34,41,46,55]. In a typical injection 3D printing experiment, the guanosine/hemin CL hydrogel (“ink”) was extruded from a medical syringe (“pen”) to write 3D-shaped patterns on a glass substrate (Figure 8a). Upon addition of a H_2_O_2_ solution, the CL emission of the 3D printed biosensor could be imaged employing a smartphone’s camera coupled with a custom dark box (Figure 8b). Therefore, the guanosine/hemin CL hydrogel has been demonstrated to be a promising candidate for syringe-injectable, flexible, and patternable biosensors and bioelectronics.

## 4. Conclusions

In this work, a simple and instrument-free biosensor for glucose measurement in serum has been developed exploiting a smartphone’s CMOS camera technology. The system is ideally suited for widespread POC applicability, as it employs a ready-to-use analytical cartridge that only requires the addition of a sample. Furthermore, unlike electrochemical glucose biosensors, no electronics, power supply, or dedicated equipment is required, other than the smartphone’s built-in camera. To this end, a CL self-assembled guanosine-derived hydrogel was prepared in the presence of hemin and luminol. Thanks to its good biocompatibility, biodegradability, optical transparency, and injectability, the guanosine/hemin CL hydrogel is ideally suited for the development of biosensors based on H_2_O_2_ detection. Upon addition of the GOx enzyme, the hydrogel could be used as responsive biomaterial for the detection of glucose. Indeed, the sol-gel interconversion of the biomaterial allowed the encapsulation of the enzyme inside the structure of the hydrogel, enhancing its stability and catalytic activity even in non-optimal conditions (i.e., in an alkaline pH environment) and generating an intense, long-lasting CL emission, which could be conveniently detected employing a smartphone’s built-in CMOS camera. An analytical cartridge and a mini dark box produced by 3D printing technology provided an easy-to-use, rapid, and sensitive analytical device that can be applied at the POC for the measurement of glucose serum levels. The analysis of glucose-spiked artificial serum samples demonstrated the selectivity and accuracy of the biosensor, which allowed measurement of both hyper- and hypoglycemic samples. This approach could be applied to other oxidases, thus enabling the development of other specific bioassays to quantify several biomarkers of clinical interest, such as cholesterol, ethanol, cortisol, and bilirubin.

## Figures and Tables

**Table 1:** Glucose concentrations measured in glucose-spiked artificial serum samples using the smartphone-based glucose biosensor and the reference enzymatic glucose colorimetric assay. ^1^

Glucose Concentration (mmol L^−1^)	Smartphone-Based CL Biosensor ^2^			Colorimetric Reference Method ^2^		
	Found (mmol L^−1^)	Recovery (%)	RSD (%)	Found (mmol L^−1^)	Recovery (%)	RSD (%)
0.5	0.55 ± 0.05	110	9.1	0.45 ± 0.10	90	22.2
3.0	3.25 ± 0.15	108	4.6	2.90 ± 0.15	97	5.2
5.0	4.80 ± 0.30	96	6.3	5.25 ± 0.40	105	7.6
6.5	6.00 ± 0.50	92	8.3	6.50 ± 0.50	100	7.7
8.0	8.20 ± 1.00	103	12.2	7.40 ± 0.65	93	8.8
10.0	10.7 ± 0.80	107	7.4	9.45 ± 0.55	95	5.8

^1^ Samples were analyzed after 1:4 (*v/v*) dilution with PBS. ^2^ Each datum represents the mean ± SD of three replicates.

## Data Availability

Data will be available upon request.
